# Artificial Intelligence Adoption in Public Health Practice: A Cross-Sectional Study of Practical Determinants Among Healthcare Professionals

**DOI:** 10.3390/healthcare14162465

**Published:** 2026-08-10

**Authors:** Carla Aurelia Stoiacovici, Adrian Cosmin Ilie, Felicia Marc, Silviu Brad, Alina Doina Tanase, Horia Silviu Branea

**Affiliations:** 1 Doctoral School, “Victor Babeș” University of Medicine and Pharmacy Timișoara, 300041 Timișoara, Romania; carla.stoiacovici@umft.ro; 2 Division of Public Health and Management, Department III Functional Sciences, “Victor Babeș” University of Medicine and Pharmacy Timișoara, 300041 Timișoara, Romania; ilie.adrian@umft.ro; 3 Department of Medical Sciences, Faculty of Medicine and Pharmacy, University of Oradea, 410073 Oradea, Romania; 4 Department II, Radiology and Medical Imaging, General and Dento-Maxillary Imaging, Faculty of Dental Medicine, “Victor Babeș” University of Medicine and Pharmacy Timișoara, Eftimie Murgu Square 2, 300041 Timișoara, Romania; 5 Department of Professional Legislation in Dental Medicine, Faculty of Dental Medicine, “Victor Babeș” University of Medicine and Pharmacy Timișoara, Eftimie Murgu Square No. 2, 300041 Timișoara, Romania; tanase.alina@umft.ro; 6 Research Centre in Dental Medicine Using Conventional and Alternative Technologies, Faculty of Dental Medicine, “Victor Babeș” University of Medicine and Pharmacy Timișoara, Eftimie Murgu Square 2, 300041 Timișoara, Romania; 7 Department of Internal Medicine I—Medical Semiotics II, “Victor Babeș” University of Medicine and Pharmacy Timișoara, 300041 Timișoara, Romania; branea.horia@umft.ro

**Keywords:** artificial intelligence, public health, health personnel, cross-sectional studies, data protection

## Abstract

Background and objectives: Artificial intelligence (AI) is increasingly embedded in public health workflows, yet adoption among practitioners remains uneven and is shaped by knowledge, legal awareness, and operational barriers. This cross-sectional study characterised determinants of AI adoption among healthcare professionals and examined how legal concern moderates the translation of technical knowledge into practical use, at a single Romanian tertiary academic centre. Methods: We surveyed 93 healthcare professionals (physicians, nurses, public health specialists, residents) at the “Pius Brînzeu” Clinical Emergency County Hospital and “Victor Babeș” University of Medicine and Pharmacy Timișoara. Participants were classified as AI adopters or non-adopters. Likert-derived composite scores (0–100; Cronbach’s α 0.79–0.88) quantified knowledge, trust, legal concern, privacy concern, and workflow confidence. Group comparisons used independent-samples *t*-tests and χ^2^ tests; associations used Pearson correlation; predictors of adoption and usage intensity were modelled with logistic and multiple linear regression; a two-way ANOVA tested profession-by-training effects. Significance was set at *p* < 0.05. Benjamini–Hochberg false-discovery-rate correction was applied across the 18 bivariate tests reported in this study, and adjusted q-values are reported alongside unadjusted *p*-values. Results: Adopters (n = 51) were younger (34.7 ± 7.5 vs. 43.2 ± 8.5 years; *p* < 0.001) and reported higher knowledge (67.3 vs. 48.6; *p* < 0.001) and workflow confidence (64.2 vs. 41.9; *p* < 0.001) but lower legal concern (58.4 vs. 71.2; *p* < 0.001). Knowledge correlated positively with usage intensity (r = 0.536; *p* < 0.001), whereas legal concern correlated negatively (r = −0.426; *p* < 0.001). In multivariable models, younger age (OR = 0.91; *p* = 0.004), knowledge (OR = 1.06; *p* = 0.005), and trust (OR = 1.07; *p* = 0.005) independently predicted adoption. The linear model explained 46.1% of usage variance. Stratified analysis suggested legal concern attenuated the knowledge–usage slope (β: 0.51→0.18); however, the formal knowledge-by-concern interaction term was not statistically significant (*p* = 0.191), and this pattern is therefore exploratory. Conclusions: In this modest, single-centre sample, AI adoption was independently associated with knowledge and trust, and legal concern was independently and negatively associated with usage intensity; the apparent dampening of the knowledge–usage relationship by legal concern was suggestive but not statistically confirmed. Targeted legal-regulatory literacy and structured training may support practical AI uptake in public health settings, pending confirmation in larger, multicentre studies.

## 1. Introduction

The integration of artificial intelligence (AI) into clinical and public health practice has accelerated over the past decade, moving from experimental decision-support prototypes to operational tools embedded in triage, surveillance, diagnostic imaging, and population health analytics [1]. As these systems migrate from research environments into routine workflows, the determinants of their real-world adoption have become a pressing scientific question. Public health, in particular, occupies a distinctive position because it operates at the intersection of individual care, population-level data stewardship, and statutory accountability, meaning that the conditions enabling or impeding AI adoption are unlikely to mirror those described in purely clinical specialties [2].

A substantial body of literature has examined healthcare professionals’ attitudes toward AI, generally reporting cautious optimism tempered by concerns about reliability, explainability, and the erosion of professional autonomy [3,4]. However, much of this work treats knowledge and attitude as the principal levers of adoption, implicitly assuming that better-informed and more trusting practitioners will inevitably use AI tools more intensively. This assumption neglects the regulatory environment in which European practitioners operate, where data protection and emerging AI-specific legislation impose obligations that may either reassure or deter potential adopters depending on how well those frameworks are understood [5].

The legal dimension of AI adoption remains comparatively understudied at the level of individual practitioner behaviour. While institutional governance and medico-legal liability have been discussed extensively in commentary and policy literature [6,7], empirical data linking practitioners’ legal awareness to their actual usage patterns are scarce. Existing quantitative surveys have measured clinicians’ trust, perceived usefulness, and general ethical concern [8,9,10], and legal-ethical analyses have mapped liability and accountability questions at a conceptual level [11,12], but, to our knowledge, no prior study has simultaneously quantified individual-level legal awareness and subjective legal concern and related both to a measured behavioural indicator of AI use. It is plausible that legal concern functions not as a simple binary deterrent but as a moderator that reshapes how other determinants, such as technical knowledge, translate into practice. A knowledgeable professional who perceives high legal exposure may rationally restrain their use of AI, even when technically capable of deploying it effectively [13].

Practical and operational barriers constitute a third, partially independent axis. Time constraints, inadequate training, immature infrastructure, and uncertainty about how to validate AI outputs all shape whether favourable attitudes convert into sustained behaviour [11,14]. These barriers are frequently cited but rarely disentangled from knowledge and legal factors, leaving open the question of which constraints are most amenable to intervention. In resource-constrained or transitional health systems, such as those in Central and Eastern Europe, these operational realities may dominate the adoption calculus in ways that differ from high-resource settings [15].

The Romanian context offers an instructive case study. As a European Union member state, Romania is subject to the full European regulatory architecture, yet its health system faces particular pressures relating to workforce distribution, digital infrastructure maturity, and continuing professional education [16]. Tertiary academic centres such as those in Timișoara serve simultaneously as clinical hubs, public health reference institutions, and training environments, making them ideal sites in which to observe how knowledge, legal awareness, and practical constraints interact across a heterogeneous professional population.

Against this background, the present cross-sectional study pursues three objectives. First, to characterise and compare AI adopters and non-adopters across demographic, cognitive, legal, and operational dimensions. Second, to identify independent predictors of both binary adoption and continuous usage intensity using multivariable modelling. Third, and most distinctively, to test whether legal concern moderates the relationship between technical knowledge and practical AI use. By foregrounding the legal-moderation hypothesis, this study seeks to contribute novel evidence beyond the well-established knowledge–attitude paradigm. The intended contribution is threefold: the explicit measurement of legal constructs, distinguishing objective awareness from subjective concern, at the level of the individual practitioner; their linkage to a continuous behavioural indicator of AI use rather than to attitudes alone; and their examination within the regulatory context created by the General Data Protection Regulation (GDPR) and the EU AI Act in a Central and Eastern European health system. Three hypotheses were prespecified: (H1) technical knowledge and trust are positively associated with AI adoption and usage intensity; (H2) perceived legal concern is negatively associated with usage intensity independently of knowledge and trust; and (H3) legal concern moderates, specifically attenuates, the association between knowledge and usage intensity.

## 2. Materials and Methods

### 2.1. Study Design and Setting

This was a single-centre, analytical cross-sectional study conducted at the “Pius Brînzeu” Clinical Emergency County Hospital Timișoara in collaboration with the “Victor Babeș” University of Medicine and Pharmacy Timișoara, Romania. The institution functions as a tertiary referral centre and an academic training environment, hosting a diverse professional population spanning clinical specialties, public health practice, and academic medicine. Data were collected over a defined twelve-week enrolment window. The cross-sectional design was selected because the primary aim was to characterise the prevailing distribution of AI adoption determinants at a single point in time, rather than to establish temporal precedence or causal direction, which would have required a longitudinal or interventional architecture. The study is reported in accordance with the STROBE (Strengthening the Reporting of Observational Studies in Epidemiology) recommendations for cross-sectional studies, and the completed checklist is provided as Supplementary Material (Table S1).

The target population comprised healthcare professionals with at least six months of active practice who could plausibly encounter AI-enabled tools in their workflow. Eligible categories included attending physicians, nurses, public health specialists, and senior residents. We excluded purely administrative staff, professionals on extended leave, and individuals whose roles involved no clinical or population-health data handling. A total of 93 participants were enrolled, a sample size constrained by the single-centre setting and the eligible workforce available during the enrolment window. A formal power assessment indicated that a sample of 93 (51 adopters, 42 non-adopters) provided at least 80% power (α = 0.05, two-tailed) to detect standardised between-group differences of d ≥ 0.59 and bivariate correlations of r ≥ 0.29, and yielded approximately ten adoption events per predictor for a five-predictor logistic model, the conventional lower bound for stable estimation. The study was, by design, not powered for interaction testing: a post hoc calculation indicated approximately 31% power to detect a knowledge-by-legal-concern interaction of the magnitude observed (f^2^ ≈ 0.02), with roughly 340 participants required to reach 80% power. All interaction and moderation analyses were therefore treated as exploratory, and their results are reported with this caveat.

### 2.2. Participants and Exposure Definition

The principal exposure-of-interest classification distinguished AI adopters from non-adopters. Adopters were operationally defined as professionals who reported deliberate, recurring use of at least one AI-enabled tool (for example, clinical decision support, automated image analysis, natural-language documentation assistants, or predictive surveillance dashboards) within the preceding three months. Non-adopters reported no such recurring use. This binary served as the dependent variable in the logistic model and as the grouping variable in bivariate comparisons. The dichotomy was retained as the primary classification for interpretability and comparability with prior adoption surveys, although it necessarily collapses heterogeneous engagement, from daily integration to occasional use. All analyses of usage additionally employed the continuous usage-intensity measure, and the frequency gradient within adopters is reported descriptively (see Section 3).

Recruitment proceeded by stratified invitation across professional categories to ensure representation of physicians, nurses, public health specialists, and residents. Each invitation was linked to a unique non-reusable token to prevent duplicate submissions. In total, 142 eligible professionals were invited; 101 (71.1%) accessed the survey platform and 95 submitted questionnaires, of which 2 fell below the pre-specified 80% item-completion threshold and were excluded, leaving 93 analysable participants and an overall response rate of 65.5% (93/142). The professional-category distribution of participants did not differ significantly from that of the invited population (χ^2^ = 0.94, *p* = 0.816), and the sex distribution was likewise comparable; no further demographic information was available for non-responders. Participation was voluntary and uncompensated, and respondents could withdraw at any point before final submission. Demographic and professional covariates collected at baseline included age, biological sex, years of professional experience, and profession category, all of which were retained as potential confounders for the multivariable stage of analysis.

### 2.3. Instruments and Variables

Cognitive and attitudinal constructs were measured using a structured questionnaire whose items were aggregated into five normalised composite scores ranging from 0 to 100: technical knowledge, trust in AI, perceived legal risk concern, data-privacy concern, and workflow-integration confidence. Each composite was derived from multiple Likert-anchored items, rescaled linearly to the 0–100 metric to facilitate cross-domain comparison and regression interpretation. The questionnaire was developed for this study by adapting item content from published surveys of professional attitudes toward AI [8,9,10,17] and from the regulatory literature on the GDPR and the EU AI Act [18,19]; each composite comprised four to six five-point Likert items. Content validity was reviewed by a small expert panel (two public health specialists, one clinician, and one specialist in health law), and the instrument was piloted on ten professionals not included in the analytic sample, leading to minor wording revisions. Internal consistency in the present sample was acceptable to good for all composites (Cronbach’s α: technical knowledge 0.87; trust in AI 0.84; legal concern 0.81; privacy concern 0.79; workflow confidence 0.88). The instrument has not undergone external validation or formal factor analysis, and the composites should be regarded as study-specific rather than established scales. A continuous measure of AI usage intensity served as the secondary outcome for linear modelling. Usage intensity was operationalised as self-reported hours of AI-enabled tool use in a typical working week, weighted by the character of that use: hours in which AI output directly informed a professional task were weighted 1.0, whereas hours of peripheral or background use (for example, passive monitoring of a predictive dashboard) were weighted 0.5, yielding weighted hours per week.

Legal awareness was additionally captured through discrete yes/no items addressing awareness of the applicability of data-protection regulation, the provisions of AI-specific legislation, liability frameworks for AI-related errors, and the existence of an institutional AI policy, enabling categorical analysis. Legal awareness and legal concern were conceptualised as distinct constructs: awareness denotes factual familiarity with the applicable regulatory instruments, whereas concern denotes subjective apprehension of legal exposure associated with AI use. Because we hypothesised that concern is partly fed by informational deficit, the association between the two constructs was tested directly. For multivariable modelling, the four awareness items were collapsed into a single dichotomous legal-awareness indicator, with participants classified as aware if they endorsed at least two of the four items. Training status was operationalised separately as self-reported completion of any formal, structured AI-related training (an institutional course, an accredited continuing-education module, or a university curriculum component), recorded as a binary variable; it is distinct from the subjective ‘inadequate training’ barrier item, which captures perceived insufficiency of preparation irrespective of formal course completion. Self-reported practical barriers (time constraints, inadequate training, infrastructure limitations, and output-validation uncertainty) were likewise recorded as binary endorsements. To minimise social desirability bias, substantive responses were collected without direct identifiers attached, and a subset of items was reverse-coded to detect inattentive responding; records flagged by these consistency checks were reviewed before inclusion.

**Figure 1:**
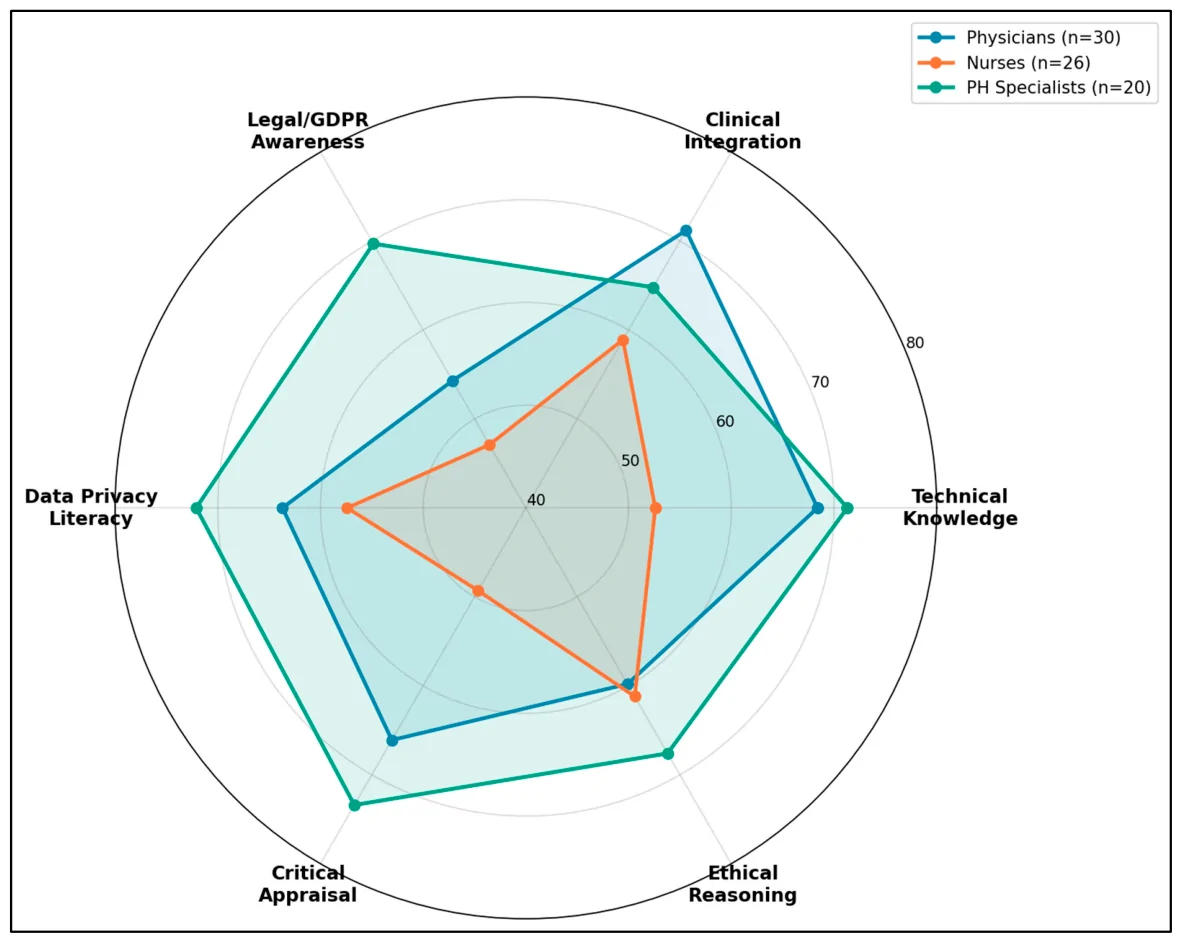
AI-competency profiles by profession. Radar plot of mean normalised scores (0–100) across six competency domains for the three professional subgroups. Residents (n = 17) are not displayed as a separate profile; the plot is restricted to the three established professional categories (displayed n = 76; see Section 2.5). Definitions, item counts, and internal-consistency coefficients for all six axes, and their correspondence with the five composite scores used in the inferential analyses, are given in Section 2.3; the three axes not used in any model (Clinical Integration, Ethical Reasoning, and Critical Appraisal) are descriptive only.

The descriptive competency profile presented in Figure 1 comprises six domains, each scored on the same normalised 0–100 metric; their correspondence with the constructs defined above is as follows. Three of the six axes are the constructs already specified: “Technical Knowledge” is the technical-knowledge composite; “Data Privacy” is the data-privacy composite; and “Legal/GDPR Awareness” is the four-item legal-awareness battery expressed as the percentage of items endorsed (Kuder–Richardson-20 = 0.74). The remaining three axes were measured by additional sets of five-point Likert items that were retained for descriptive profiling only and were not entered into any regression, ANOVA, or moderation model, and they are defined here for reproducibility. “Clinical Integration” (five items; Cronbach’s α = 0.85) captures the perceived fit of AI output with the respondent’s own diagnostic or therapeutic decision pathway, including whether AI-derived information is available at the point at which a decision is actually made. “Ethical Reasoning” (four items; Cronbach’s α = 0.80) captures self-rated capacity to identify and reason about equity, consent, transparency, and bias issues arising in AI-assisted care. “Critical Appraisal” (five items; Cronbach’s α = 0.82) captures self-rated ability to interrogate an AI output before acting on it, including recognition of training-data limitations, performance drift, and inappropriate extrapolation beyond the validated population. All three domains were developed for this study, reviewed by the same expert panel, and piloted together with the rest of the instrument; like the five principal composites, they are study-specific and have not been externally validated. Because these three domains are descriptive, they are reported in Figure 1 as group means without inferential testing, and the five composites listed at the start of this section remain the only scores used in the inferential analyses reported in Section 3.

### 2.4. Data Collection and Quality Control

Questionnaires were administered electronically through a secure institutional platform. Range and logic checks were embedded in the survey instrument to prevent out-of-bounds or internally inconsistent entries, and completion required approximately fifteen minutes. Partially completed instruments below an 80% item-completion threshold were excluded from analysis to limit the influence of non-random missingness (n = 2; 2.1% of submitted questionnaires). The few remaining missing values on continuous composites (7 item-level responses, <0.1% of all item-level data; no composite score was missing in its entirety, and no analysis lost more than two cases) were addressed by available-case analysis rather than imputation, given the low overall missingness and the analytical rather than predictive purpose of the study.

All data were exported into a de-identified analytic dataset prior to statistical processing. Data integrity was further safeguarded by independent double-entry verification of a random 20% subsample, which yielded no discrepancies exceeding pre-specified tolerance. The study was conducted in accordance with the Declaration of Helsinki, and informed consent was obtained from all participants before enrolment.

### 2.5. Statistical Analysis

Descriptive statistics summarised continuous variables as means with standard deviations and categorical variables as counts with percentages. Between-group comparisons of continuous variables used independent-samples *t*-tests, with Welch’s correction applied where variance homogeneity, assessed by Levene’s test, was not supported. Categorical comparisons used Pearson’s χ^2^ test, with attention to expected cell frequencies. Bivariate associations among continuous measures were quantified using Pearson correlation coefficients. All tests were two-tailed with significance set at *p* < 0.05. Because the bivariate comparisons described above together form a family of 18 tests (five composite-score comparisons, four legal-awareness comparisons, four practical-barrier comparisons, and five correlations), the false-discovery rate across this family was controlled using the Benjamini–Hochberg procedure, and both the unadjusted *p*-value and the Benjamini–Hochberg-adjusted q-value are reported for every test in that family. A result is described as statistically significant only where the adjusted q-value remained below 0.05; results significant at *p* < 0.05 but not at q < 0.05 are reported as directional observations and are not interpreted as established group differences. Where the more conservative Bonferroni criterion (α = 0.05/18 = 0.0028) would alter inference, this is stated explicitly in the Results and in Section 4.2. No correction was applied to the four baseline demographic comparisons, which describe sample composition rather than test study hypotheses, or to the prespecified multivariable models and the moderation analysis, which evaluate a small number of a priori hypotheses rather than constituting a screening family.

Multivariable analysis comprised three model families. A binary logistic regression model estimated independent predictors of AI adoption, reporting adjusted odds ratios (OR) with 95% confidence intervals (CI) and model discrimination via the area under the receiver-operating-characteristic curve (AUC). A multiple linear regression model estimated predictors of continuous usage intensity, reporting unstandardised and standardised coefficients alongside the coefficient of determination. A two-way analysis of variance (ANOVA) examined the joint and interactive effects of profession and training status on usage intensity. To address the principal novel hypothesis, an interaction term between knowledge and legal concern was tested and visualised through tertile-stratified regression. Standardised effect sizes (Cohen’s d) with 95% CI were computed for the composite domains. Multicollinearity in the multivariable models was assessed with variance inflation factors (VIF), with values below 5 considered acceptable. Internal validity of the logistic model was examined by bootstrap resampling (1000 replicates) to obtain an optimism-corrected AUC, and calibration was assessed with the Hosmer–Lemeshow test; a parsimonious three-predictor sensitivity model and a model substituting years of experience for age were also estimated. For the two-way ANOVA, effect sizes are reported as partial η^2^. For profession-stratified analyses, the resident category (n = 17), comprising physicians-in-training, was handled as follows: in the descriptive competency profiling of Figure 1, residents were omitted so that the three established professional groups could be characterised without the transitional heterogeneity of trainees (displayed n = 76); in the two-way ANOVA, which required the full sample, residents were merged with the attending-physician category, yielding a three-level profession factor (physicians including residents, nurses, and public health specialists; N = 93). In addition to the tertile-stratified visualisation, the full moderation model containing the knowledge-by-legal-concern product term is reported with its coefficient and 95% CI. Analyses were performed in Python version 3.11.5 (SciPy version 1.11.4 and statsmodels version 0.14.1).

## 3. Results

**Figure 2:**
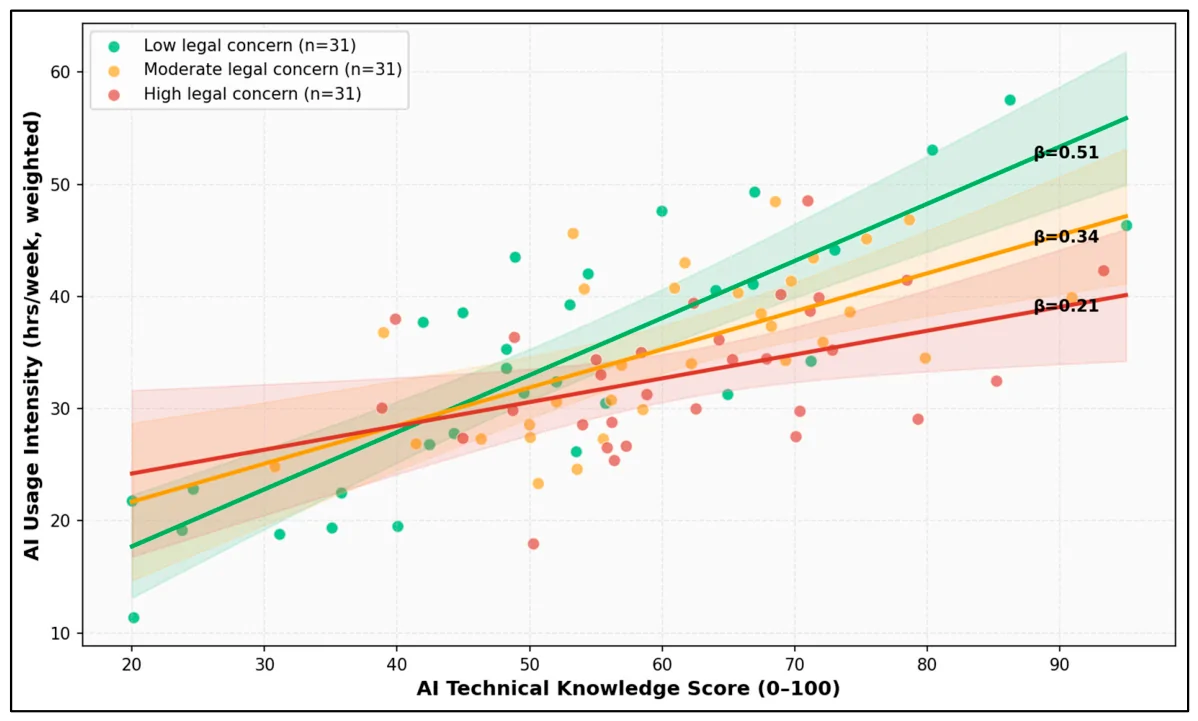
Knowledge–usage association stratified by legal-concern tertile: a descriptive, exploratory analysis. Knowledge–usage regression slopes stratified by legal-concern tertile, with 95% confidence bands. The formal knowledge-by-concern interaction term was not statistically significant (*p* = 0.191); the stratified pattern is presented as descriptive and hypothesis-generating.

**Figure 3:**
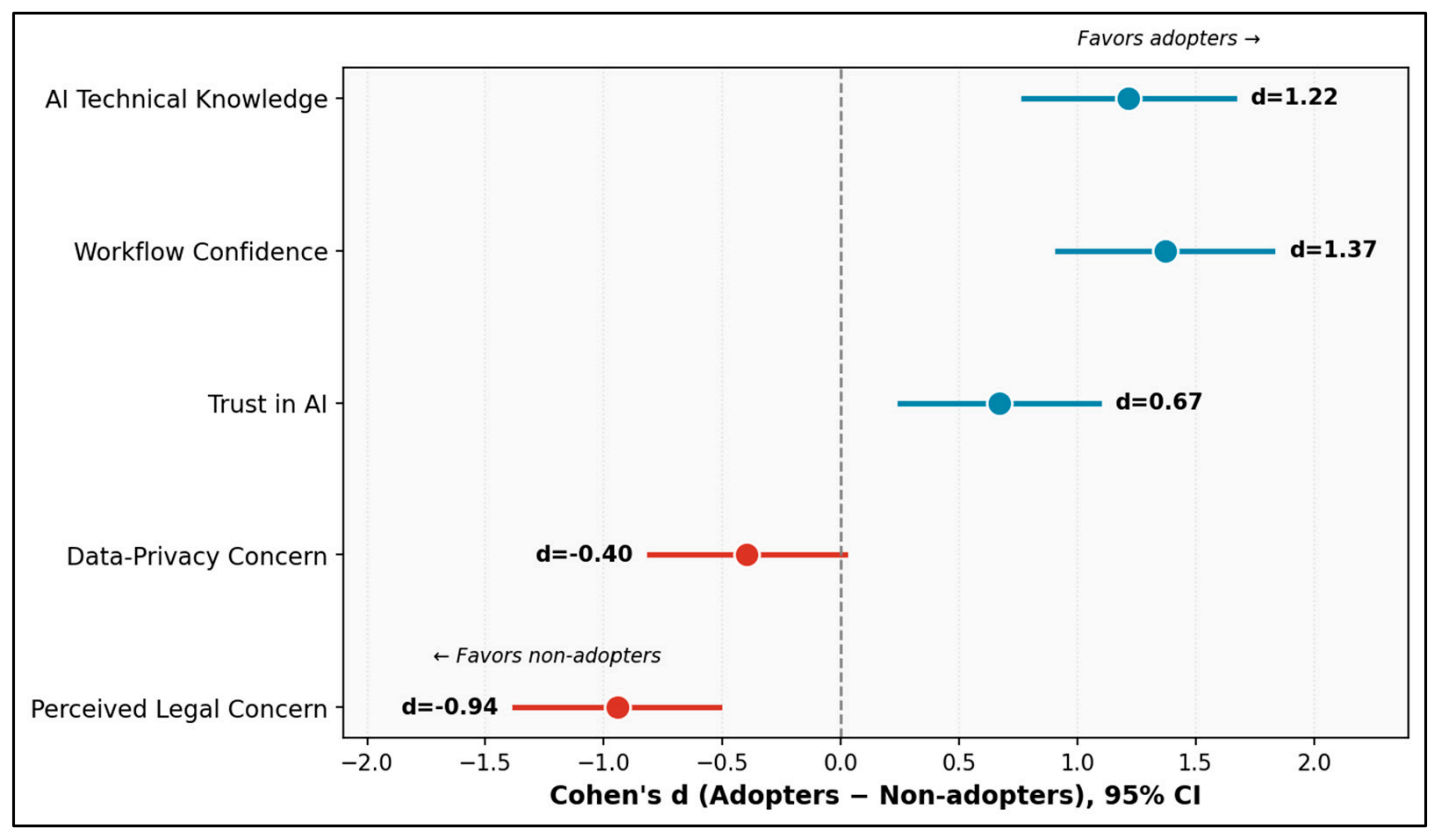
Effect sizes across composite domains. Forest plot of standardised effect sizes (Cohen’s d) with 95% confidence intervals comparing adopters with non-adopters.

**Figure 4:**
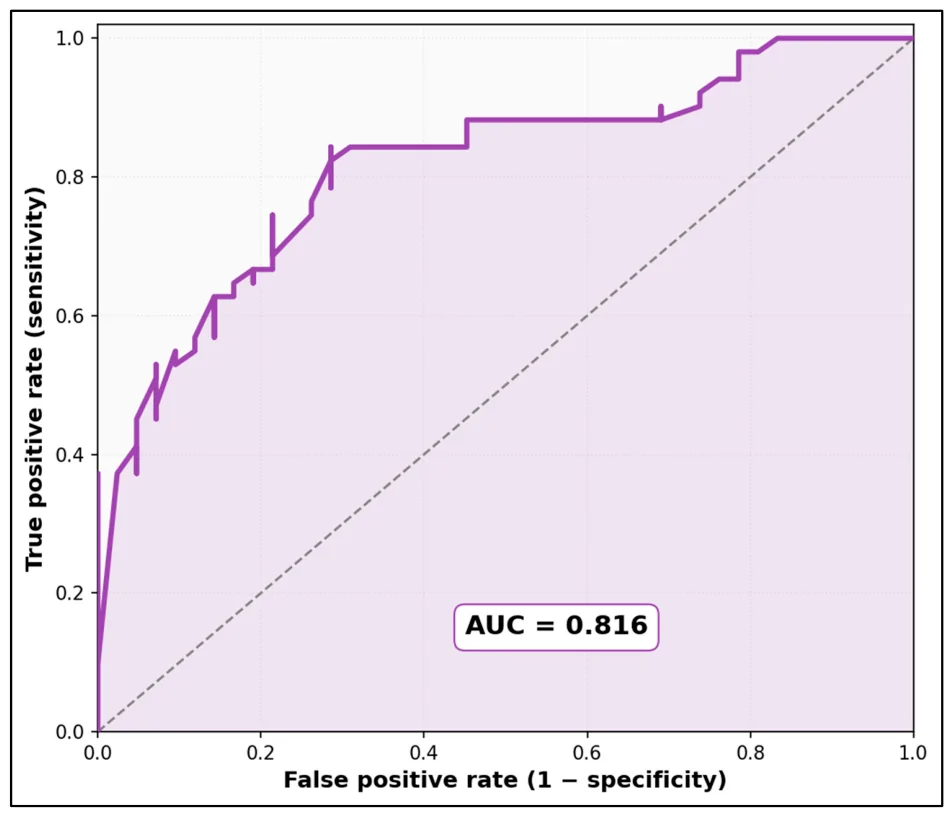
Discrimination of the adoption model. Receiver-operating-characteristic curve for the multivariable logistic model predicting AI adoption.

A total of 93 healthcare professionals were enrolled, of whom 51 (54.8%) were classified as AI adopters and 42 (45.2%) as non-adopters. The two groups, comparison contrasts, subgroup analyses, correlations, and multivariable models are presented in Figure 1, Figure 2, Figure 3 and Figure 4 and in the tables that follow.

Adopters were, on average, more than eight years younger than non-adopters (34.7 ± 7.5 versus 43.2 ± 8.5 years), a difference that reached high statistical significance (t = 4.97, *p* < 0.001; Table 1). A parallel and equally pronounced separation emerged for years of professional experience, with adopters averaging 9.4 ± 5.3 years against 16.6 ± 6.6 years among non-adopters (t = 5.71, *p* < 0.001). These two findings are mutually reinforcing and consistent with the broader observation that earlier-career professionals, who trained in more digitally saturated environments, are more receptive to integrating novel computational tools into routine practice. In contrast, the distribution of biological sex did not differ significantly between groups (54.9% versus 64.3% female; χ^2^ = 0.84, *p* = 0.360), indicating that adoption in this cohort was not patterned along sex lines. Similarly, professional category was distributed comparably across the two groups (χ^2^ = 1.69, *p* = 0.639).

Decision-support tools were the most frequently reported primary application, used by 33.3% of adopters, followed by documentation and natural-language processing assistants at 25.5%, image-analysis systems at 23.5%, and population-surveillance analytics at 17.6% (Table 2). This spread suggests, within the limits of the small numbers in each tool category, that AI adoption in this setting is not confined to a single niche such as radiological image interpretation, which has historically driven much of the clinical AI narrative; instead, adoption extends into documentation and public-health surveillance domains that are central to the institution’s dual clinical and population-health mission. The mean usage intensity of 14.7 ± 6.8 weighted hours per week indicates substantial but not saturating engagement, and the relatively wide standard deviation signals considerable heterogeneity among adopters in how deeply these tools are woven into daily practice. Self-rated proficiency averaged 62.3 ± 15.1 on the 0–100 scale, a moderately high figure that nonetheless leaves meaningful room for skill development. The frequency distribution further illuminates the texture of adoption: while 41.2% reported daily use and 45.1% weekly use, a residual 13.7% described only occasional engagement, suggesting that even within the adopter category there exists a gradient from intensive integrators to peripheral, intermittent users.

Public health specialists (n = 20) recorded the highest scores on the three domains most relevant to population-data stewardship—legal/GDPR awareness (69.7), data privacy (72.1), and critical appraisal (73.4)—consistent with their regulatory and surveillance responsibilities. Physicians (n = 30) recorded the highest score on clinical integration (71.2) and scored closely to specialists on technical knowledge (68.4 versus 71.3). Because Figure 1 domains are reported descriptively (Section 2.3), no significance testing was performed on these between-profession differences. The most consequential signal in the figure is the legal/GDPR awareness axis, where the specialist-to-nurse gap reached 22.6 points (69.7 versus 47.1), the single largest inter-group separation anywhere in the radar. Nurses (n = 26) scored lowest on most axes but achieved parity in ethical reasoning (61.2), indicating that the deficit is specific to formal legal-regulatory knowledge rather than to professional values.

Adopters demonstrated markedly higher technical knowledge (67.3 ± 14.2 versus 48.6 ± 16.7; t = 5.75, *p* < 0.001), a nearly nineteen-point gap that represents the single largest attitudinal separation in the dataset and confirms that command of AI fundamentals tracks closely with practical engagement. Trust in AI followed the same direction, with adopters scoring 61.8 ± 13.4 against 52.3 ± 15.1 among non-adopters (t = 3.18, *p* = 0.002; q = 0.005), reinforcing the intuition that confidence in the technology underpins willingness to deploy it. The most theoretically interesting reversal appears in perceived legal concern, which ran significantly higher among non-adopters (71.2 ± 14.3) than adopters (58.4 ± 12.9; t = −4.49, *p* < 0.001). This inverse pattern is central to the study’s novel hypothesis, suggesting that heightened apprehension about legal exposure accompanies abstention from AI use. Workflow-integration confidence showed the largest standardised separation of all (64.2 ± 15.3 versus 41.9 ± 17.4; t = 6.49, *p* < 0.001), implying that the practical belief in one’s ability to fit AI into daily routines is a powerful correlate of adoption. Notably, data-privacy concern did not differ significantly between groups (69.7 ± 13.1 versus 74.8 ± 12.6; t = −1.91, *p* = 0.060; q = 0.082) and remained clearly non-significant after Benjamini–Hochberg correction; it is therefore not interpreted here as a near-significant trend. The four remaining composite-score differences in Table 3 all retained significance after correction (q ≤ 0.005).

Adopters were nominally more likely to be aware of GDPR applicability to AI systems (80.4% versus 61.9%; χ^2^ = 3.91, *p* = 0.048), but this difference did not survive correction for multiple testing (q = 0.072) and is therefore reported as a directional observation rather than an established group difference (Table 4). Adopters were substantially more likely to know the provisions of the EU AI Act (64.7% versus 33.3%; χ^2^ = 9.07, *p* = 0.003; q = 0.005), the strongest categorical separation in this table and one of only two awareness items to remain significant after correction. They were also more familiar with their own institution’s AI policy (47.1% versus 21.4%; χ^2^ = 6.61, *p* = 0.010; q = 0.017), the second item to retain significance after correction. This configuration is conceptually important when read alongside Table 3: although non-adopters reported higher subjective legal concern, adopters possessed greater objective legal awareness on the two items that remained significant after correction for multiple testing. The apparent paradox dissolves if one interprets concern as partly a function of uncertainty rather than knowledge; those who understand the regulatory framework may feel better equipped to navigate it and therefore less anxious, whereas non-adopters may experience diffuse apprehension precisely because they are less informed about the rules governing AI use. Consistent with this interpretation, the number of awareness items endorsed correlated inversely with the legal-concern composite across the full sample (r = −0.31, *p* = 0.002; this supplementary correlation falls outside the corrected family of Table 3, Table 4, Table 5 and Table 6 and is reported unadjusted); this observation is, however, correlational and cannot establish whether concern reflects informational deficit rather than, for example, greater generalised risk aversion among non-adopters or overconfidence among adopters. Awareness of the liability framework for AI-related errors showed the weakest separation and did not reach significance under either the unadjusted or the adjusted criterion (43.1% vs. 26.2%; χ^2^ = 2.89, *p* = 0.089; q = 0.107), though the trend favoured adopters and the relatively low absolute awareness in both groups signals a shared blind spot.

The standout finding is inadequate training, reported by 81.0% of non-adopters but only 52.9% of adopters (χ^2^ = 8.01, *p* = 0.005; q = 0.008), making it the only barrier to reach statistical significance after correction for multiple testing and identifying training deficits as a primary and potentially modifiable impediment to adoption (Table 5). Infrastructure limitations followed a similar directional pattern, cited by 61.9% of non-adopters versus 43.1% of adopters, but fell short of significance both before and after correction (χ^2^ = 3.25, *p* = 0.072; q = 0.092), suggesting infrastructure may contribute secondarily without being decisive in this particular setting. Strikingly, the two most universally endorsed barriers, time constraints and output-validation uncertainty, did not differ significantly between groups. Time constraints were reported by 60.8% of adopters and 69.0% of non-adopters (χ^2^ = 0.69, *p* = 0.407), and output-validation uncertainty by 68.6% and 78.6% respectively (χ^2^ = 1.16, *p* = 0.282). Because older professionals may endorse the training barrier simply because their formal education predates widespread AI integration, this association was re-examined in relation to age: endorsement of the training barrier increased with age (Spearman ρ = 0.29, *p* = 0.005), yet in an age-adjusted logistic model the barrier remained significantly associated with non-adoption (OR = 2.98, 95% CI 1.12–7.93, *p* = 0.029), indicating that the training deficit is not wholly reducible to generational effects (Figure 2).

Figure 2 presents the exploratory stratified analysis addressing the moderation hypothesis (H3). The slope linking technical knowledge to usage intensity steepens monotonically as legal concern falls: β = 0.18 in the high-concern tertile, β = 0.38 in the moderate tertile, and β = 0.51 in the low-concern tertile, a near-threefold attenuation from low to high concern. Descriptively, the pattern suggests that knowledge may convert into actual AI use more readily when legal apprehension is low; among highly concerned professionals, the regression line remained almost flat across the knowledge range. The significant negative correlation between legal concern and usage (r = −0.426, *p* < 0.001) and the independent negative coefficient for legal concern in the linear model (standardised β = −0.254, *p* = 0.002) confirm legal concern as an independent negative correlate of usage, but they do not, by themselves, establish moderation. In the full moderation model, the knowledge-by-legal-concern product term was not statistically significant (B = −0.0031, 95% CI −0.0078 to 0.0016; *p* = 0.191), and the study had limited power (approximately 31%) to detect an interaction of this magnitude (Section 2.1). The stratified pattern should therefore be read as a suggestive, hypothesis-generating observation requiring replication in larger samples, not as a confirmed moderation effect.

The strongest single association links technical knowledge to usage intensity (r = 0.536, *p* < 0.001), a moderate-to-strong positive correlation indicating that roughly 29% of the variance in how intensively professionals use AI is shared with their level of technical knowledge. Trust in AI also correlated positively with usage intensity (r = 0.382, *p* < 0.001), confirming that attitudinal confidence accompanies behavioural engagement, though somewhat less strongly than knowledge does. The legal-concern correlation provides the most consequential result for the study’s novel angle: legal concern was negatively and significantly associated with usage intensity (r = −0.426, *p* < 0.001), meaning that greater apprehension about legal exposure accompanies measurably reduced AI use. This inverse relationship is robust and of comparable magnitude to the positive trust correlation, lending quantitative weight to the argument that the legal dimension deserves equal standing with the knowledge and trust dimensions in models of AI adoption. Intriguingly, age showed essentially no linear correlation with usage intensity among the full cohort (r = −0.035, *p* = 0.739; q = 0.739). The four remaining correlations in Table 6 retained significance after Benjamini–Hochberg correction (q < 0.001) and also satisfied the more conservative Bonferroni criterion, making them the most statistically robust bivariate findings in the study.

Each additional year of age reduced the adjusted odds of adoption by approximately 9% (OR = 0.91, 95% CI 0.86–0.97, *p* = 0.004), confirming that the generational gradient observed in Table 1 survives adjustment for cognitive and attitudinal factors and is not simply a proxy for them (Table 7). Both knowledge and trust retained independent significance, with each one-point increase on their respective 0–100 scales raising adoption odds by roughly 6% (OR = 1.06, *p* = 0.005) and 7% (OR = 1.07, *p* = 0.005) respectively; their joint retention despite their bivariate intercorrelation indicates that each captures non-redundant explanatory variance. Notably, prior training showed no independent effect once knowledge was in the model (OR = 1.05, *p* = 0.929), which is interpretable rather than contradictory: the pattern is consistent with, although it does not formally demonstrate, a mediating pathway in which training operates through the knowledge it imparts; no formal mediation analysis (for example, a bootstrapped indirect-effect estimate) was performed, and this interpretation remains tentative. Legal awareness, significant in bivariate analysis, attenuated to non-significance after adjustment (OR = 2.44, 95% CI 0.81–7.32, *p* = 0.111), though its point estimate of more than doubled odds and its wide confidence interval suggest a possible true effect that this modest sample was underpowered to confirm. The overall model fit was satisfactory, with a significant likelihood-ratio test, a pseudo-R^2^ near 0.42, and acceptable discrimination (AUC = 0.816). Bootstrap internal validation (1000 resamples) yielded an optimism-corrected AUC of 0.79, and calibration was adequate (Hosmer–Lemeshow χ^2^ = 6.74, df = 8, *p* = 0.565). Variance inflation factors were below 2 for all predictors (maximum 1.63), indicating that the moderate intercorrelation between knowledge and trust did not destabilise coefficient estimation; nonetheless, with approximately ten events per predictor, the wide confidence intervals of the binary predictors reflect imprecise estimation, and the Nagelkerke pseudo-R^2^ is not directly comparable to a linear-model R^2^ and should be interpreted only as a relative fit index. A parsimonious sensitivity model retaining only age, knowledge, and trust yielded nearly identical discrimination (AUC = 0.804), supporting the robustness of the three significant predictors. In a further sensitivity analysis substituting years of experience for age, experience showed a comparable inverse association with adoption (OR = 0.90 per year, 95% CI 0.84–0.96, *p* = 0.003). Because age and experience were strongly collinear (r = 0.87), they were not modelled simultaneously, and the age effect is best interpreted as an early-career effect that this design cannot separate from experience.

The linear regression extends the analysis from the binary adoption decision to the graded question of how intensively AI is used, and it delivers the study’s most quantitatively informative model, explaining 46.1% of adjusted variance in usage intensity (F-test *p* < 0.001; Table 8). All four predictors achieved independent significance, and the standardised coefficients permit an approximate ranking of relative predictive contribution, although, given the conceptual and empirical intercorrelation of the constructs, they should not be read as fully independent effects. Knowledge was the strongest predictor (standardised β = 0.468, *p* < 0.001), meaning that a one-standard-deviation rise in knowledge corresponds to nearly half a standard deviation increase in usage intensity, the strongest effect in the model. Workflow-integration confidence ranked second (standardised β = 0.387, *p* < 0.001), reaffirming that the practical belief in one’s capacity to embed AI in daily routines translates powerfully into actual usage volume. Trust contributed a moderate independent effect (standardised β = 0.314, *p* < 0.001). Critically, legal concern entered the model as a significant negative predictor (unstandardised β = −0.197, standardised β = −0.254, *p* = 0.002). Variance inflation factors in the linear model ranged from 1.28 to 1.71, well below conventional thresholds, indicating that multicollinearity did not materially destabilise the coefficient estimates.

The main effect of profession was significant (F = 15.99, *p* < 0.001), establishing that baseline usage intensity differs meaningfully across physicians, nurses, and public health specialists, independent of whether they have received training (Table 9). This profession effect, which was masked in the binary adoption comparison of Table 1, surfaces here because intensity is a more granular outcome than the adopt/non-adopt dichotomy and is better able to register the distinct workflow demands of different roles. The main effect of training status was likewise significant and of comparable magnitude (F = 17.32, *p* < 0.001), confirming that trained professionals use AI more intensively than untrained ones, consistent with the barrier analysis in Table 5 that flagged training deficits as the decisive practical obstacle. The interaction term did not reach statistical significance (profession × training: F = 1.21, *p* = 0.303; partial η^2^ = 0.027, 90% CI 0.00–0.09). Its absence cannot be taken as confirmation of the null hypothesis: the study was underpowered for interaction tests, and the data are compatible with, but do not prove, a training association of similar magnitude across professional roles.

Figure 3 expresses the adopter–non-adopter contrasts on a standardised metric, allowing the magnitude of group differences to be compared directly across domains irrespective of their original scales. Two domains showed large positive effects favouring adopters: workflow confidence (d = 1.37) and technical knowledge (d = 1.22), both exceeding the conventional threshold for a large effect and confirming these as the dominant discriminators of adoption. Trust in AI showed a moderate positive effect (d = 0.67). On the opposing side, perceived legal concern produced the largest negative effect (d = −0.94), a near-large effect favouring non-adopters that quantifies, in standardised terms, how strongly legal apprehension separates the two groups and reinforces its status as the study’s key constraint variable. Data-privacy concern (d = −0.40) was the only domain whose confidence interval crossed zero, mirroring its non-significant *t*-test (*p* = 0.060; q = 0.082).

Figure 4 summarises the classificatory performance of the multivariable logistic adoption model as a whole. The receiver-operating-characteristic curve bows well above the diagonal line of chance, yielding an area under the curve of 0.816, which falls in the conventionally “good-to-excellent” range and indicates that the combined predictor set—age, knowledge, trust, training, and legal awareness—discriminates adopters from non-adopters with high reliability. Bootstrap internal validation indicated that this apparent discrimination is modestly optimistic (optimism-corrected AUC = 0.79; see the commentary to Table 7). In practical terms, a randomly selected adopter has roughly an 82% probability of receiving a higher model-predicted adoption score than a randomly selected non-adopter.

## 4. Discussion

### 4.1. Analysis of Findings

This cross-sectional study of 93 healthcare professionals identified a coherent set of determinants distinguishing AI adopters from non-adopters and, more distinctively, identified legal concern as an independent and quantitatively meaningful negative correlate of AI usage. The principal bivariate findings—that adopters are younger, more knowledgeable, more trusting, and more workflow-confident—align with the established adoption literature and lend the cohort face validity [3,4]. Prior surveys of clinician attitudes have repeatedly emphasised the centrality of trust and perceived usefulness [8,9,10], and our standardised effect sizes reinforce that workflow confidence and knowledge carry the largest discriminating weight. The departure from prior work lies in the consistent and robust role of the legal dimension, which surfaced as a significant negative correlate of usage intensity (r = −0.426), retained independent significance in multivariable linear regression (standardised β = −0.254), and appeared, in exploratory stratified analysis, to shape the knowledge–usage relationship, although the formal interaction term was not statistically significant (*p* = 0.191). Whereas governance frameworks have been discussed conceptually [11,14,18], few empirical studies have linked individual legal perception directly to measured behaviour, and our data argue that models of AI adoption confined to knowledge and attitude are incomplete [5,13]. At the same time, it must be underlined that the central moderation hypothesis (H3) was not statistically confirmed; the evidence for the legal dimension rests on robust bivariate and multivariable associations with usage, not on a demonstrated interaction.

A particularly instructive tension emerged between objective legal awareness and subjective legal concern, which moved in opposite directions: adopters knew more about GDPR, the EU AI Act, and institutional policy yet worried less [12,18,19]. One interpretation of this pattern is that legal concern in this population is fed substantially by informational deficit rather than by accurate appraisal of genuine regulatory risk, echoing observations that uncertainty about liability and accountability dampens clinical enthusiasm for AI [12,13]. This interpretation is, however, speculative and was not directly tested: the study measured neither the accuracy of participants’ risk appraisals nor the specificity of their concerns. Alternative explanations merit equal consideration: adopters’ lower concern could reflect overconfidence born of familiarity with the technology rather than accurate appraisal, and non-adopters’ apprehension may represent diffuse, generalised risk aversion expressed in legal terms because legal questions are salient in current public discourse, rather than genuinely regulatory anxiety. The inverse correlation between awareness and concern (r = −0.31, *p* = 0.002) is consistent with the informational-deficit account but cannot adjudicate among these possibilities. If apprehension partly reflects uncertainty rather than knowledge, the intervention implied is educational rather than regulatory; equipping professionals with concrete understanding of the applicable frameworks may simultaneously raise awareness and lower the diffuse anxiety that suppresses usage [20,21]. The low absolute awareness of liability frameworks in both groups identifies a shared blind spot that responsible-AI guidance and critical-appraisal work have repeatedly flagged as critical for safe deployment [15,22,23], and our findings give that concern an empirical, behaviour-linked grounding rather than a purely normative one.

The practical-barrier and ANOVA results converge on training as the most actionable lever. Inadequate training was the single barrier that significantly discriminated non-adopters from adopters, training status exerted a significant main effect on usage intensity, and the absence of a detectable profession-by-training interaction was compatible with training benefits accruing similarly across professional roles, although the study was underpowered for interaction tests, consistent with implementation-science accounts that identify capability gaps as a dominant obstacle to clinical AI uptake [24,25]. This combination is encouraging for implementation because it suggests that a single, well-designed training programme could raise AI usage across physicians, nurses, and public health specialists alike without bespoke tailoring [26,27]. When paired with the legal-literacy findings, the evidence points toward an integrated curriculum that couples technical competency development with explicit regulatory education, addressing both the knowledge driver and the legal-concern constraint in a single intervention—an approach increasingly advocated for trustworthy and patient-centred AI deployment [17,28,29] and especially pertinent to digitally maturing health systems such as Romania’s [16,30]. Concretely, the findings point to five curriculum components: (i) supervised, hands-on practice with the tool classes actually deployed institutionally (decision support, documentation assistants, image analysis, and surveillance analytics); (ii) structured instruction in output validation and critical appraisal, addressing the validation uncertainty endorsed by more than two-thirds of respondents; (iii) applied legal-regulatory literacy covering the practical obligations arising from the GDPR and the EU AI Act; (iv) explicit teaching of liability allocation for AI-assisted decisions, the domain with the lowest awareness in both groups; and (v) orientation to the institution’s own AI policy, of which fewer than half of adopters and only a fifth of non-adopters were aware. Because the training main effect was significant while profession did not detectably modify it, such a curriculum could plausibly be delivered as a single cross-professional programme complemented by role-specific practical modules.

### 4.2. Study Limitations

Several limitations temper the interpretation of these findings. First, the cross-sectional design precludes causal inference; although knowledge, trust, and legal concern are associated with adoption and usage, the temporal ordering cannot be established, and reverse causation—whereby usage itself cultivates knowledge and reduces concern—remains plausible. If reverse causation operates, the observed associations would partly reflect consequences rather than antecedents of adoption, and the cross-sectional coefficients would overstate the prospective value of intervening on knowledge or concern; only longitudinal designs can resolve this. Second, the single-centre setting at one Romanian tertiary academic institution limits external generalisability, as adoption dynamics may differ in primary-care, rural, or non-academic environments and across health systems with divergent regulatory cultures. This limitation deserves particular emphasis: the Romanian setting combines specific regulatory, resource, workforce, and cultural characteristics, including a digitally maturing infrastructure and centralised tertiary academic care, and the findings should not be extrapolated to primary-care networks, rural facilities, non-academic hospitals, or health systems outside Romania without direct verification. Relatedly, the sample’s professional composition does not mirror institutional staffing: physicians and residents together outnumbered nurses in the sample, whereas nurses substantially outnumber physicians in the hospital’s actual workforce, so nursing perspectives are under-represented relative to the institution’s staffing structure. Third, the sample size of 93, while adequate for the principal bivariate and main-effect analyses, constrained statistical power for the interaction and moderation tests; the knowledge-by-legal-concern interaction did not reach significance (*p* = 0.191) despite a directionally consistent stratified pattern, so the moderation hypothesis remains suggestive rather than confirmed, with post hoc power of approximately 31% for the observed interaction (Section 2.1). Fourth, reliance on self-reported usage and self-rated proficiency introduces potential recall and social-desirability bias, only partially mitigated by anonymised aggregate reporting and consistency checks. Because all constructs and both outcomes were self-reported within a single instrument, common method variance may have inflated the observed intercorrelations, including those between knowledge, trust, and usage intensity; a post hoc Harman single-factor test indicated that the first unrotated factor accounted for 31% of the variance, below the conventional 50% threshold, which is reassuring but does not exclude method effects. No objective measure of AI knowledge (for example, a scored competency test) or of usage (for example, system log data) was available, and self-reports may not correspond to objective ability or behaviour; furthermore, because AI adoption is currently perceived as innovative and desirable, self-reported usage may be inflated by social desirability. Fifth, the composite scores, including the three descriptive competency domains reported only in Figure 1, were internally structured but were not externally validated against an established instrument, and the binary adopter definition inevitably collapses a continuum of engagement. In addition, although the response rate was moderate (65.5%) and the professional-category distribution of responders matched that of the invited population, non-response bias cannot be excluded: professionals interested in AI may have been more likely to participate, and only limited demographic information was available for non-responders. Sixth, the study reports a substantial number of bivariate comparisons relative to its sample size, so inflation of the type I error rate is a material concern. The false-discovery rate across the 18 tests of Table 3, Table 4, Table 5 and Table 6 was controlled using the Benjamini–Hochberg procedure, and one result that was nominally significant at *p* < 0.05, awareness of GDPR applicability to AI (*p* = 0.048; q = 0.072), did not survive correction and is accordingly no longer interpreted as a group difference; the borderline data-privacy comparison (*p* = 0.060; q = 0.082) likewise remained non-significant. Under the still more conservative Bonferroni criterion (α = 0.0028), awareness of an institutional AI policy (*p* = 0.010) and the inadequate-training barrier (*p* = 0.005) would additionally lose significance, while the differences in trust (*p* = 0.002) and EU AI Act awareness (*p* = 0.003) would be retained only marginally. The findings robust to every correction strategy applied are the adopter–non-adopter differences in technical knowledge, workflow-integration confidence, and perceived legal concern, together with the correlations of knowledge, trust, and legal concern with usage intensity; the training-barrier result, although sensitive to Bonferroni adjustment, is additionally supported by the age-adjusted logistic model and by the significant training main effect in the two-way ANOVA. The remaining bivariate results should be regarded as provisional and in need of replication in an adequately powered sample. Finally, residual confounding cannot be excluded, and the observational nature of the data means unmeasured factors such as institutional incentives or peer influence may contribute to the observed associations.

## 5. Conclusions

This cross-sectional investigation of AI adoption among 93 healthcare professionals at a Romanian tertiary academic centre yields three principal conclusions. First, AI adoption and usage intensity were robustly and independently associated with technical knowledge, trust, and workflow-integration confidence, with younger professionals significantly more likely to adopt; these determinants survived multivariable adjustment and jointly explained a substantial share of variance in both adoption (pseudo-R^2^ ≈ 0.42; AUC = 0.816) and usage intensity (adjusted R^2^ = 0.461). Second, and most distinctively, perceived legal concern emerged as an independent negative correlate of AI usage, an association that persisted after adjustment for knowledge, trust, and workflow confidence; stratified analysis suggested, without statistically confirming (interaction *p* = 0.191), that legal concern may attenuate the efficiency with which technical knowledge converts into practice. The opposing directions of objective legal awareness and subjective legal concern are consistent with the interpretation, which remains speculative, that this apprehension is fed more by informational uncertainty than by accurate risk appraisal; alternative explanations, including generalised risk aversion among non-adopters and overconfidence among adopters, cannot be excluded. Third, inadequate training emerged as the most clearly modifiable practical barrier, associated with non-adoption independently of age and robust to false-discovery-rate correction, with no detectable variation in the training association across professional roles. All of these conclusions are associative in nature: the cross-sectional design precludes causal inference, the modest, single-centre sample limits generalisability, and the number of bivariate comparisons required correction for multiple testing, after which the difference in GDPR-applicability awareness was no longer statistically significant. Collectively, these findings argue for an integrated workforce-development strategy that pairs technical competency training with explicit regulatory and legal-literacy education, thereby addressing simultaneously the strongest positive driver and the principal perceptual constraint on AI uptake. Multicentre, longitudinal, and ideally interventional studies are warranted to confirm the moderation hypothesis and to establish causal pathways.

## Figures and Tables

**Table 1:** Baseline demographic and professional characteristics by adoption status. Continuous variables compared by independent-samples *t*-test; categorical variables by Pearson χ^2^ test.

Characteristic	Adopters (n = 51)	Non-Adopters (n = 42)	Test Statistic	*p*-Value
Age, years (mean ± SD)	34.7 ± 7.5	43.2 ± 8.5	t = 4.97	<0.001
Female sex, n (%)	28 (54.9)	27 (64.3)	χ^2^ = 0.84	0.360
Experience, years (mean ± SD)	9.4 ± 5.3	16.6 ± 6.6	t = 5.71	<0.001
Profession, n (%)			χ^2^ = 1.69	0.639
Physician	19 (37.3)	11 (26.2)		
Nurse	12 (23.5)	14 (33.3)		
Public health specialist	11 (21.6)	9 (21.4)		
Resident	9 (17.6)	8 (19.0)		

**Table 2:** AI usage characteristics among adopters (n = 51), summarised as counts with percentages or means with standard deviations.

Usage Characteristic	n (%) or Mean ± SD
Primary tool: decision support	17 (33.3)
Primary tool: documentation/NLP	13 (25.5)
Primary tool: image analysis	12 (23.5)
Primary tool: surveillance analytics	9 (17.6)
Usage intensity, weighted hrs/week	14.7 ± 6.8
Self-rated proficiency (0–100)	62.3 ± 15.1
Frequency: daily use, n (%)	21 (41.2)
Frequency: weekly use, n (%)	23 (45.1)
Frequency: occasional use, n (%)	7 (13.7)

**Table 3:** Knowledge, trust, and concern composite scores (0–100) by adoption status, compared by independent-samples *t*-test. q denotes the Benjamini–Hochberg false-discovery-rate-adjusted *p*-value computed across the 18 bivariate tests reported in Table 3, Table 4, Table 5 and Table 6 (Section 2.5).

Composite Score (0–100)	Adopters (n = 51)	Non-Adopters (n = 42)	t	*p*-Value	q (BH-Adjusted)
AI technical knowledge	67.3 ± 14.2	48.6 ± 16.7	5.75	<0.001	<0.001
Trust in AI	61.8 ± 13.4	52.3 ± 15.1	3.18	0.002	0.005
Perceived legal concern	58.4 ± 12.9	71.2 ± 14.3	−4.49	<0.001	<0.001
Data-privacy concern	69.7 ± 13.1	74.8 ± 12.6	−1.91	0.060	0.082
Workflow-integration confidence	64.2 ± 15.3	41.9 ± 17.4	6.49	<0.001	<0.001

**Table 4:** Legal-awareness items by adoption status (proportion answering “yes”), compared by Pearson χ^2^ test. q denotes the Benjamini–Hochberg-adjusted *p*-value across the 18 bivariate tests of Table 3, Table 4, Table 5 and Table 6 (Section 2.5).

Legal-Awareness Item (Aware: Yes)	Adopters n (%)	Non-Adopters n (%)	χ^2^	*p*-Value	q (BH-Adjusted)
GDPR applicability to AI	41 (80.4)	26 (61.9)	3.91	0.048	0.072
EU AI Act provisions	33 (64.7)	14 (33.3)	9.07	0.003	0.005
Liability framework for AI errors	22 (43.1)	11 (26.2)	2.89	0.089	0.107
Institutional AI policy	24 (47.1)	9 (21.4)	6.61	0.010	0.017

**Table 5:** Self-reported practical barriers to AI use by adoption status, compared by Pearson χ^2^ test. q denotes the Benjamini–Hochberg-adjusted *p*-value across the 18 bivariate tests of Table 3, Table 4, Table 5 and Table 6 (Section 2.5).

Barrier Reported (Yes)	Adopters n (%)	Non-Adopters n (%)	χ^2^	*p*-Value	q (BH-Adjusted)
Time constraints	31 (60.8)	29 (69.0)	0.69	0.407	0.430
Inadequate training	27 (52.9)	34 (81.0)	8.01	0.005	0.008
Infrastructure limitations	22 (43.1)	26 (61.9)	3.25	0.072	0.092
Output-validation uncertainty	35 (68.6)	33 (78.6)	1.16	0.282	0.317

**Table 6:** Pearson correlations among continuous study variables (n = 93), with 95% confidence intervals. q denotes the Benjamini–Hochberg-adjusted *p*-value across the 18 bivariate tests of Table 3, Table 4, Table 5 and Table 6 (Section 2.5).

Variable Pair	r	95% CI	*p*-Value	q (BH-Adjusted)
Knowledge and Usage intensity	0.536	0.37 to 0.67	<0.001	<0.001
Trust and Usage intensity	0.382	0.19 to 0.55	<0.001	<0.001
Legal concern and Usage intensity	−0.426	−0.59 to −0.24	<0.001	<0.001
Age and Usage intensity	−0.035	−0.24 to 0.17	0.739	0.739
Knowledge and Trust	0.486	0.31 to 0.63	<0.001	<0.001

**Table 7:** Multivariable binary logistic regression predicting AI adoption. Likelihood-ratio test *p* < 0.001; Nagelkerke pseudo-R^2^ ≈ 0.42; model discrimination AUC = 0.816.

Predictor	Adjusted OR	95% CI	*p*-Value
Age (per year)	0.91	0.86–0.97	0.004
Knowledge (per point)	1.06	1.02–1.11	0.005
Trust (per point)	1.07	1.02–1.11	0.005
Prior training (yes vs no)	1.05	0.34–3.25	0.929
Legal awareness (yes vs no)	2.44	0.81–7.32	0.111

**Table 8:** Multiple linear regression predicting AI usage intensity (n = 93). Model R^2^ = 0.484; adjusted R^2^ = 0.461; overall F-test *p* < 0.001.

Predictor	β (Unstd.)	SE	Std. β	t	*p*-Value
Knowledge	0.275	0.046	0.468	5.96	<0.001
Trust	0.214	0.053	0.314	4.06	<0.001
Legal concern	−0.197	0.062	−0.254	−3.19	0.002
Workflow confidence	0.253	0.051	0.387	4.97	<0.001
Constant	13.863	5.851	—	2.37	0.020

**Table 9:** Two-way analysis of variance (ANOVA) of usage intensity by profession and training status, with the profession-by-training interaction term. Profession was modelled as a three-level factor in which residents were merged with the physician category (Section 2.5). Partial η^2^: profession 0.269; training status 0.166; interaction 0.027.

Source	Sum of Squares	df	F	*p*-Value
Profession	2727.27	2	15.99	<0.001
Training status	1477.58	1	17.32	<0.001
Profession × Training	206.47	2	1.21	0.303
Residual	7420.45	87	—	—

## Data Availability

The data presented in this study are available on request from the corresponding author due to ethical restrictions.

## Supplementary Materials

The following supporting information can be downloaded at: https://www.mdpi.com/article/10.3390/healthcare14162465/s1. Table S1: completed STROBE checklist for cross-sectional studies, indicating the manuscript section in which each reporting item is addressed.
