# Supplementary material for: Artificial Intelligence Adoption in Public Health Practice: A Cross-Sectional Study of Practical Determinants Among Healthcare Professionals
Source: Healthcare (Basel). 2026 Aug 10;14(16):2465. doi: 10.3390/healthcare14162465 (PMC13511971; doi:10.3390/healthcare14162465)
Supplement: Supplementary file 1 [file healthcare-14-02465-s001.zip › healthcare-4445087-supplementary.pdf]

Supplementary Material

Table S1. Completed STROBE checklist for cross-sectional studies, indicating the manuscript section in which each reporting item is addressed. Manuscript: "Artificial Intelligence Adoption in Public Health Practice: A Cross-Sectional Study of Practical Determinants Among Healthcare Professionals" (healthcare-4445087).

Item	Recommendation topic	STROBE requirement (abbreviated)	Location in manuscript
1	Title and abstract	Indicate the study design in the title or abstract; provide an informative, balanced summary.	Title; Abstract (design stated; results and limitations summarised)
2	Background / rationale	Explain the scientific background and rationale.	Introduction, paragraphs 1–5
3	Objectives	State specific objectives, including any prespecified hypotheses.	Introduction, final paragraph (objectives and hypotheses H1–H3)
4	Study design	Present key elements of study design early in the paper.	Section 2.1
5	Setting	Describe the setting, locations, and relevant dates.	Section 2.1 (institution; twelve-week enrolment window)
6	Participants	Give eligibility criteria and the sources and methods of selection.	Sections 2.1–2.2 (eligibility, exclusions, stratified invitation)
7	Variables	Clearly define all outcomes, exposures, predictors, and potential confounders.	Section 2.3 (composites, awareness items, barriers, training status, usage intensity)
8	Data sources / measurement	For each variable, give sources of data and details of methods of assessment.	Sections 2.3–2.4 (questionnaire development, pilot, Cronbach's α, electronic administration)
9	Bias	Describe any efforts to address potential sources of bias.	Sections 2.3 (reverse-coded items, anonymised responses), 2.4 (double entry), 4.2
10	Study size	Explain how the study size was arrived at.	Section 2.1 (power assessment; detectable effect sizes; interaction power)
11	Quantitative variables	Explain how quantitative variables were handled; describe groupings.	Sections 2.3 and 2.5 (0–100 rescaling; tertiles; dichotomies; resident merging)
12	Statistical methods	Describe all statistical methods, subgroups, missing data, and sensitivity analyses.	Sections 2.4–2.5 (tests, models, VIF, bootstrap, calibration, sensitivity analyses)

Item	Recommendation topic	STROBE requirement (abbreviated)	Location in manuscript
13	Participants (flow)	Report numbers at each stage (invited, examined, analysed) and reasons for non-participation.	Section 2.2 (142 invited; 101 accessed; 95 submitted; 2 excluded; 93 analysed)
14	Descriptive data	Give characteristics of participants and information on missing data.	Table 1; Section 2.4 (missing-data quantification)
15	Outcome data	Report numbers of outcome events or summary measures.	Results, Tables 2–5 (adoption counts, usage intensity, awareness, barriers)
16	Main results	Give unadjusted and adjusted estimates with precision (95% CI).	Tables 6–9; Figures 2–4 (correlations; adjusted OR and β with 95% CI)
17	Other analyses	Report other analyses done (subgroups, interactions, sensitivity).	Results (tertile stratification, interaction model, age/experience and parsimonious sensitivity models, age-adjusted barrier analysis)
18	Key results	Summarise key results with reference to study objectives.	Section 5 (Conclusions)
19	Limitations	Discuss limitations, sources of potential bias or imprecision, and their direction and magnitude.	Section 4.2
20	Interpretation	Give a cautious overall interpretation considering objectives, limitations, and other evidence.	Sections 4.1 and 5 (associative framing; exploratory moderation)
21	Generalisability	Discuss the generalisability (external validity) of the results.	Section 4.2 (single-centre Romanian setting; settings excluded from extrapolation)
22	Funding	Give the source of funding and the role of the funders.	Funding statement (back matter)
